# P-1248. Phase 2 Pharmacokinetics and Anti-Drug Antibody Results of the Investigational Twice-Yearly HIV-1 Treatment Regimen Lenacapavir, Teropavimab, and Zinlirvimab

**DOI:** 10.1093/ofid/ofaf695.1439

**Published:** 2026-01-11

**Authors:** Nan Zhang, Jianmin Li, Hui Liu, Kwad Mponponsuo, Sean E Collins, Yanan Zheng

**Affiliations:** Gilead Sciences, Inc., Foster City, CA, USA, Foster City, CA; Gilead Sciences, Inc., Foster City, CA, USA, Foster City, CA; Gilead Sciences, Inc., Foster City, CA, USA, Foster City, CA; Gilead Sciences, Inc., Foster City, CA, USA, Foster City, CA; Gilead Sciences, Inc., Foster City, CA, USA, Foster City, CA; Gilead Sciences, Inc., Foster City, CA, USA, Foster City, CA

## Abstract

**Background:**

Teropavimab (TAB, GS-5423) and zinlirvimab (ZAB, GS-2872) are broadly neutralizing antibodies (bNAbs) under investigation as a twice-yearly (Q6M) combination treatment with lenacapavir (LEN) for people with HIV-1 (PWH). An ongoing Phase 2 study (NCT05729568) reported efficacy and safety of Q6M LEN, TAB, and ZAB in virologically suppressed (VS) PWH consistent with the standard of care; here, we report Week (W) 52 pharmacokinetics (PK), anti-drug antibody (ADA), and neutralizing antibody (NAb) responses.

**Figure 1.**
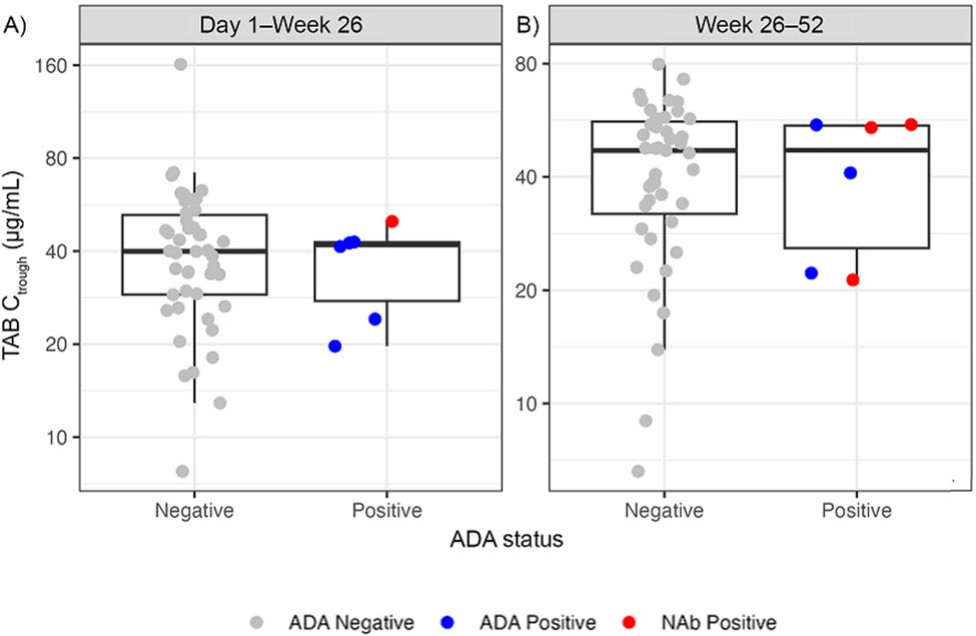
Distribution of Ctrough by ADA status of TAB at Day 1–Week 26 (A) and Week 26–52 (B) ADA-positive refers to any antibody that binds to the drug. NAb-positive are a subset of ADA-positive, which refers specifically to ADAs that are neutralizing. Box plots show the interquartile range; the bold central line represents the median. ADA, anti-drug antibody; Ctrough, trough concentration; NAb, neutralizing antibody; TAB, teropavimab.

**Figure 2.**
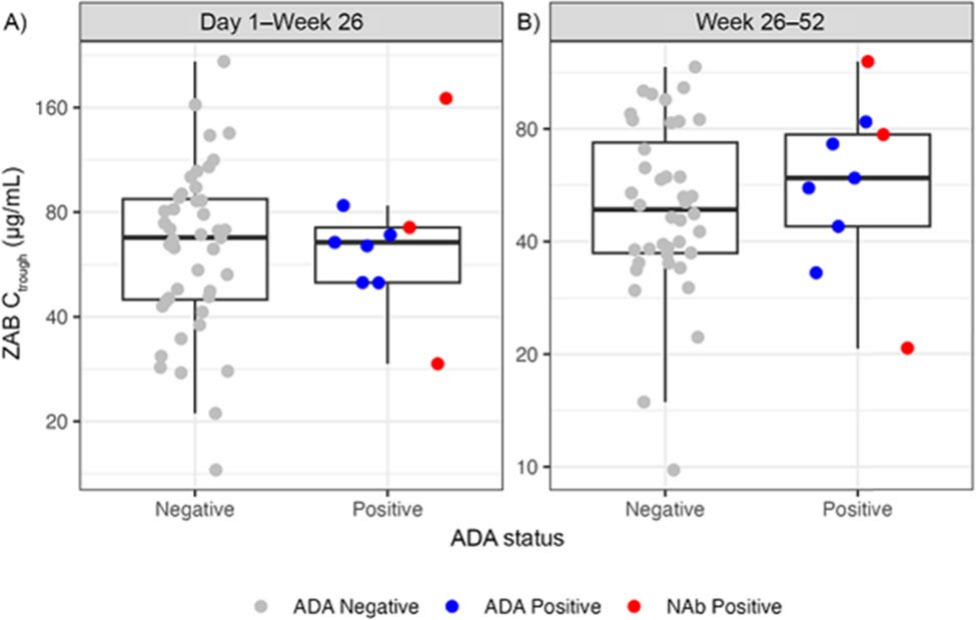
Distribution of Ctrough by ADA status of ZAB at Day 1–Week 26 (A) and Week 26–52 (B) ADA-positive refers to any antibody that binds to the drug. NAb-positive are a subset of ADA-positive, which refers specifically to ADAs that are neutralizing. Box plots show the interquartile range; the bold central line represents the median. ADA, anti-drug antibody; Ctrough, trough concentration; NAb, neutralizing antibody; ZAB, zinlirvimab.

**Methods:**

PK serum samples of TAB and ZAB and plasma samples of LEN were tested with validated electrochemiluminescence (ECL) immunoassay and liquid chromatography-mass spectrometry methods, respectively. Non-compartmental analyses based on LEN, TAB, and ZAB concentration data were conducted with WinNonLin software. Samples were tested for ADAs, with NAb assessment on ADA-positive samples, using validated ECL-based assays.

**Results:**

Fifty-three participants received LEN, TAB, and ZAB. Half-lives of TAB and ZAB were 63.5 and 89.1 days, respectively. Limited accumulation was observed after the second dose of TAB and ZAB at W26. After the Day 1 subcutaneous (SC) LEN dose (plus oral loading on Days 1+2), mean (90% CI) trough concentration (C_trough_) was 20.2 ng/mL (17.8; 22.7) at W26 (n=52); after the second SC LEN dose at W26, mean (90% CI) C_trough_ was 25.8 ng/mL (22.3; 29.3) at W52 (n=46). LEN concentrations were above the 4-fold inhibitory quotient (IQ4; 15.5 ng/mL) for wild-type HIV-1 at both timepoints. Emergent ADAs against TAB and ZAB were generally low in titer and detected in 6 (11.3%) and 9 (17.0%) participants, respectively; of these, 3 (5.7%) had emergent NAbs against TAB and ZAB, individually. All ADAs were treatment-induced; 2 (3.8%) and 6 (11.3%) participants had persistent ADAs against TAB and ZAB, respectively. There was no impact of ADAs or NAbs on the PK of TAB (Figure 1) or ZAB (Figure 2).

**Conclusion:**

Mean therapeutic concentrations of LEN, TAB, and ZAB were maintained at W52, with no evidence of drug accumulation over time. There was a low incidence of ADAs/NAbs against TAB and ZAB, and titers were generally low. No association was observed between assay-positive ADAs/NAbs and PK. LEN, TAB, and ZAB had prolonged half-lives, supporting Q6M dosing in VS PWH.

**Disclosures:**

Nan Zhang, PhD, Exelixis: Employee|Gilead science, inc.: Employee|Gilead science, inc.: Stocks/Bonds (Public Company) Jianmin Li, MS, Gilead Sciences, Inc.: Employee Hui Liu, PhD, Gilead Sciences, Inc.: Employee|Gilead Sciences, Inc.: Stocks/Bonds (Private Company) Kwad Mponponsuo, MD, MSc, Gilead Sciences, Inc.: Employee and shareholder Sean E. Collins, MD, MS, Gilead Sciences, Inc: Employee Yanan Zheng, PhD, Gilead Sciences, Inc.: Employee|Gilead Sciences, Inc.: Stocks/Bonds (Public Company)

